# Pennsylvania

**Published:** 1896-07

**Authors:** 


### LEGISLATION.

### PENNSYLVANIA.

*An Ideal Ordinance Regulating the Milk-supply.* An ordinance prohibiting the sale of adulterated, unwholesome, or impure milk in the city of Pittsburg; regulating the sale and traffic in milk; providing for the licensing of persons, firms, or corporations dealing therein; the making of examinations and tests of animals producing milk, and fixing penalties for violations of this ordinance.

SECTION 1. *Be it ordained and enacted by the city of Pittsburg in Select and Common Councils assembled, and it is hereby ordained and enacted by the authority of the same, that* whoever by himself, or by his servant or agent or as the servant or agent of any person, firm, or corporation, sells, exchanges, or delivers, or has in his or their custody or possession with intent to sell, exchange, or deliver, or exposes or offers for sale as pure milk, any milk from which the cream or any part thereof has been removed, or which has been adulterated, or changed in any respect by the addition of water or other substance, shall be liable to the penalties hereinafter provided in this ordinance.

SEC. 2. No dealer in milk and no servant or agent of such dealer, firm, or corporation shall sell, exchange, or deliver, or have in his or their custody or possession with intent to sell, exchange, or deliver, milk from which the cream or any part thereof have been removed, unless, in a conspicuous place above the centre, on the outside of each vessel, can, or package, from or in which such milk is sold, conveyed, or delivered, the words "Skimmed Milk" are permanently soldered in metallic letters not less than one inch in length, the said can, vessel, or package to be painted or japanned a bright blue color. Whoever violates the provisions of this section shall be liable to the penalties hereinafter provided in this ordinance.

SEC. 3. No person, firm, or corporation shall sell, exchange, or deliver, or have in his or their custody or possession with intent to sell, exchange, or deliver, skimmed milk containing less than nine (9) per cent. of the milk solids, exclusive of butter fat. Whoever violates the provisions of this section shall be liable to the penalties hereinafter provided in this ordinance.

SEC. 4. That every person, firm, or corporation who shall sell or offer for sale, or who shall transport or carry for the purpose of sale, or shall have in his or their possession with intent to sell, any impure, adulterated, or unwholesome milk, and every person, firm, or corporation who shall adulterate milk, or who shall keep animals for the production of milk in a crowded or unhealthy condition, or unsanitary premises, or feed the same on food that produces impure, diseased, or unwholesome milk, or who shall feed said animals on distillery waste, usually called "swill," or upon any substance in a state of putrefaction or rottenness, or upon any other substance of an unwholesome nature, or who shall not allow said animals free movement in the open air at pasture at least six hours each day, shall be liable to the penalties provided in this ordinance.

SEC. 5. That the addition of water, ice, or any other substance or thing to milk is hereby declared to be an adulteration, and milk that is obtained from animals that are fed on distillery waste, usually called "swill," or upon any substance in a state of putrefaction or rottenness, or upon any substance of an unwholesome nature, or milk that has been exposed to or is infected by the emanations, discharges, or exhalations from persons or animals having any contagious disease by which the health or life of any person or animal may be endangered, or milk from tuberculous animals, or animals suffering from any febrile disease, is hereby declared to be impure and unwholesome.

SEC. 6. No person, firm, or corporation shall sell, exchange, or deliver, or transport, or have in his, her, or their possession for the purpose of sale any milk which contains more than eighty-seven and fifty (87.50) one hundredths per centum of water and less fat than three (3) per centum, and the specific gravity of which at sixty (60) degrees Fahrenheit is not between one and twenty-nine (1.029) one thousandths and one and thirty-three (1.033) one thousandths, and all milk of lower grade or quality than is established by this section shall be deemed and taken and is hereby declared to be adulterated and impure within the meaning of this ordinance.

SEC. 7. On and after the expiration of thirty days from the passage and approval of this ordinance, for all milk brought into or offered for sale in the city of Pittsburg, satisfactory evidence must be furnished to the Bureau of Health, by the producers or dealers in said milk at their own cost and expense, that said milk has been produced by healthy animals, and especially that they are free from tuberculosis; which conditions of health shall be determined by examinations and tuberculin-tests to be made by a veterinarian who is satisfactory to the State Live-stock Sanitary Board and the Superintendent of the Bureau of Health. After said examinations and tests have been made the veterinarian shall place upon each animal found by him to be in a healthy condition an ear tag, to be furnished by the Bureau of Health, and also furnish to said bureau a certificate, upon a form to be issued by it, setting forth that each of said animals, describing the same, is free from disease, is being properly fed, and that the premises occupied by them are in good sanitary condition, which examinations and tests shall be required and certificates furnished regarding each and every additional animal purchased or secured by said producers, thereafter. Subsequent examinations, tests, and certificates as aforesaid may be required by the superintendent of said bureau whenever in his opinion, based upon reliable information, any of said animals are in an unhealthy condition or the premises occupied by them are in an unsanitary state.

SEC. 8. The Superintendent of the Bureau of Health shall, on or before the first day of September of each year, license all persons, firms, or corporations who convey milk in wagons or otherwise for the purpose of selling the same within the city of Pittsburg, said license to be renewed annually. He shall also keep a record of their names, residences, places of business, number of wagons or other vehicles used for the purpose, and the number of the license. The latter, together with the name of the owner, and the number of the wagon or vehicle shall be legibly painted on each outer side of all wagons or vehicles used in the conveyance or sale of milk, in letters not less than two inches in height. Said superintendent shall also annually license and register every person, firm, or corporation selling or offering milk for sale in a store, stand, or market-place within the city, which license shall be displayed conspicuously in said place of business. The dealer or vender shall upon the written order of the Superintendent of the Bureau of Health pay into the city treasury the sum of one dollar and receive therefor a receipt, presentation of which at the office of the Bureau of Health shall entitle said person, firm, or corporation to a license; provided that all the provisions and requirements of this ordinance have been complied with.

SEC. 9. Every person, firm, or corporation engaged or desiring to engage in the sale of milk as aforesaid shall make written application to the Bureau of Health, upon blank forms to be furnished by said bureau, asking that a license be issued, authorizing the same.

SEC. 10. That any person, firm, or corporation who shall engage in or continue the sale of milk in said city without first having obtained such license or who shall violate or fail to comply with any of the provisions of this ordinance shall be liable to a penalty not exceeding fifty (50) dollars for the first offense, and a penalty of fifty (50) dollars for a second or any subsequent offense, to be sued for in the corporate name of the city of Pittsburg, and recovered in the manner provided for the recovery of debts or penalties of like amount.

SEC. 11. In addition to the penalties mentioned and provided in the foregoing section, the Superintendent of the Bureau of Health may, by and with the consent and approval of the Director of the Department of Public Safety, revoke the license issued to the said person, firm, or corporation so offending, which license shall not be renewed or reissued during a period of one year thereafter.

SEC. 12. That any ordinance or part of ordinance conflicting with the provisions of this ordinance be, and the same is hereby repealed so far as the same affects this ordinance.

